# Using Implementation Science to Inform Workforce and Service Development in Youth Mental Health: An Australian Case Study

**DOI:** 10.1007/s43477-022-00058-z

**Published:** 2022-09-29

**Authors:** Isabel Zbukvic, Jennifer Nicholas, Craig Hamilton, Paula Cruz-Manrique, Caroline Crlenjak, Rosemary Purcell

**Affiliations:** 1 Orygen, Parkville, Victoria Australia; 2 Centre for Youth Mental Health, The University of Melbourne, Parkville, Victoria Australia

**Keywords:** Framework, Adolescent, Adolescent health services, Mental health services, Staff development, Health personnel

## Abstract

Globally, mental illness and substance use disorders are the leading cause of disability and disease burden for young people. Orygen is an Australian youth mental health organisation with a mission to reduce the impact of mental ill health on young people, families and society, through research, clinical services, advocacy, and the design and delivery of youth mental health workforce and service development initiatives. Orygen is one of only a few known research and clinical centres with a dedicated knowledge translation division, which concentrates on growing the capacity of the systems, services, and professionals who support young people experiencing mental ill health. This paper provides a case study of the workforce development team within the Orygen knowledge translation, outlining how implementation science informs their work and how the division has adapted its model in the face of COVID-19. Since 2017, the team has delivered training to more than 4000 youth mental health workers across Australia, on the topics of trauma, psychosis, mood and anxiety disorders, brief interventions, cognition and other areas of youth mental health. The COVID-19 pandemic generated abrupt and dramatic changes to the delivery of workforce and service development initiatives in Australia due to significant restrictions to travel and in-person events. It also placed major delivery demands on youth mental health services. This paper outlines how the team at Orygen adapted their approach to youth mental health workforce development in response to COVID-19, offering reflections and future directions for implementation science that can support flexible models of support in a changing system.

## Introduction

Mental illness and substance use disorders are reported as the leading cause of disability and disease burden worldwide for young people (Erskine et al., 2015). Global epidemiological data indicate that half of all lifetime mental disorders begin by 18 years of age, and 62.5% by the mid-20 s (Solmi et al., 2021) which includes mood, substance use, eating, psychotic and personality disorders. Mental ill health experienced during this important developmental period is associated with high rates of school failure, difficulties with employment, and poor social functioning, which can lead to patterns of disadvantage and dysfunction that persist into adulthood. Access to effective, evidence-based mental health care for young people is therefore critical to ensuring positive long-term mental health and functional outcomes over the lifetime (McGorry et al., 2007).

Developing the capacity of systems that support youth mental health is a global priority, and countries across the world have focused on building workforces and services needed to achieve this (Wainberg et al., 2017). In Australia, the recent Royal Commission into Victoria’s Mental Health System (2018) identified key recommendations for ‘workforce capabilities and professional development’ while other state-based Mental Health Commissions have developed strategic frameworks to support the growth and development of appropriately skilled and qualified workers to deliver high-quality interventions and programmes to the community (Mental Health Commission, 2020; NSW Health, 2018). Orygen is an Australian youth mental health organisation with a mission to reduce the impact of mental ill health on young people, their families and society through research, clinical services, advocacy, and the design and delivery of youth mental health workforce and service development initiatives. Orygen is one of only a few known research and clinical centres with a dedicated knowledge translation division, which concentrates on growing the capacity of the systems, services, and workforces who support young people with emerging and existing mental ill health. The division achieves this through education, training, consultation, coaching, and other initiatives delivered in youth mental health care settings.

From early 2020, the COVID-19 pandemic generated abrupt and dramatic changes to systems and lives globally. This paper describes how Orygen has supported youth mental health workforce development over the last five years (from 2017), with reflections on how it adapted its model in the face of COVID-19. The first section provides background context about knowledge translation in youth mental health, including Orygen’s approach to knowledge translation through youth mental health workforce development. The second section describes how implementation science supports Orygen’s workforce development activities and outputs. The third section outlines how the team has adapted its approach to the delivery of youth mental health workforce development and implementation support. By providing reflections on how implementation science has supported workforce development initiatives in response to the pandemic, this paper aims to provide insights into how implementation science is shaping adaptation in child- and family-serving services and systems and offer suggestions for future research and practice that can further develop the fields.

## Knowledge Translation in Youth Mental Health

**Fig. 1:**
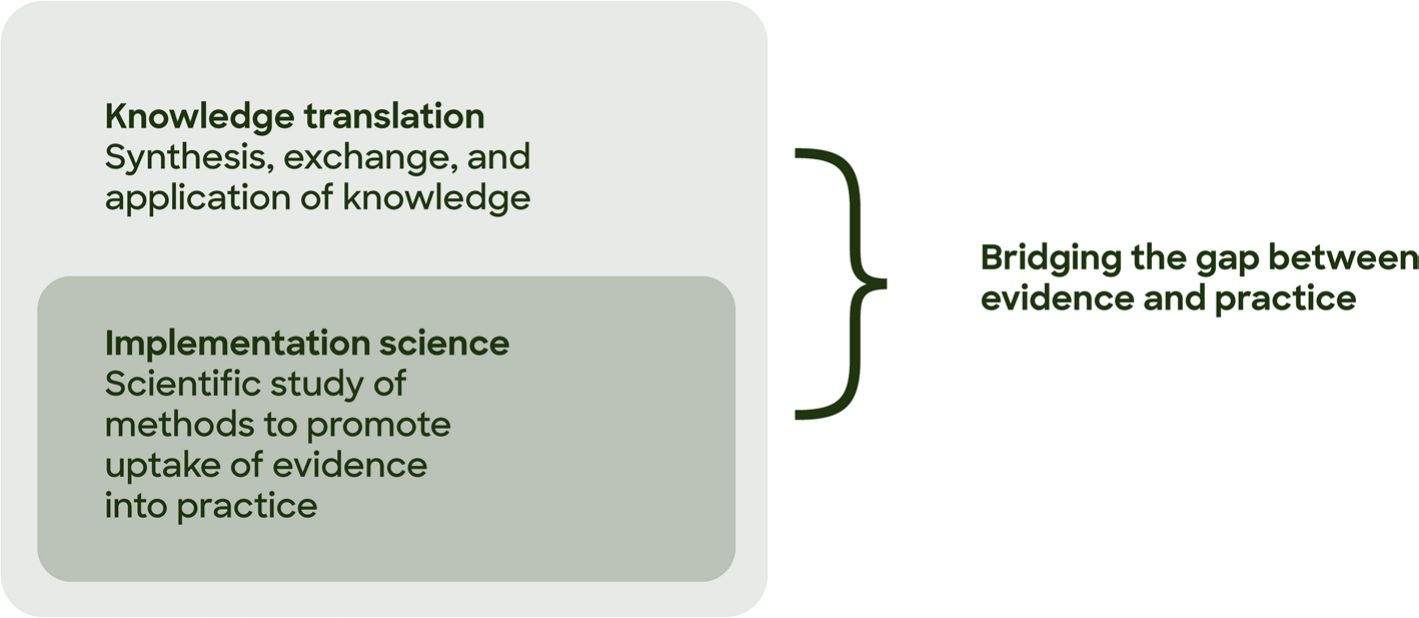
Overlap between implementation science and knowledge translation (Eccles & Mittman, 2006; Pablos‐Mendez & Shademani, 2006; Eccles & Mittman, 2006)

Knowledge translation is defined as 'the synthesis, exchange, and application of knowledge by relevant stakeholders to accelerate the benefits of global and local innovation in strengthening health systems and improving people’s health’ (Pablos‐Mendez & Shademani, 2006; Wensing & Grol, 2019). Implementation science is broadly defined as the scientific study of methods to promote the uptake of evidence into practice (Wensing & Grol, 2019). Implementation science has been described as a sub-specialty of knowledge translation (Eccles & Mittman, 2006; Esmail et al., 2020), while others define knowledge translation as a set of activities encompassed by implementation science (Mitchell & Chambers, 2017). What’s clear is that knowledge translation and implementation science are related and overlapping fields—see Fig. 1. Both knowledge translation and implementation science have focused largely on improving health, particularly through improving the quality and effectiveness of systems, services and care. Both emerged with the aim of bridging the gap between evidence and practice (Straus et al., 2009) and the increasing interest in implementation science as it relates to mental health services for children and young people has been attributed to the growing field of knowledge translation (Fixsen et al., 2005).

In the field of youth mental health, knowledge translation has gathered momentum over the last two decades, with a growing recognition of the need to build understandings of the most supported approaches for promoting the implementation of evidence-based practices and sustainable practice change (Barwick et al., 2012). Although the amount of research in youth mental health has increased exponentially in recent years, youth mental health has a history of not providing strongly supported practices and treatments (Hoagwood et al., 2001). As evidence in the area continues to proliferate, the need grows for strategies and tools to help translate research so that it may be applied in practice. Knowledge translation provides a broad range of frameworks, tools, methods and measures to understand and apply research evidence to support practice change (Straus et al., 2009).

### Orygen’s Approach to Knowledge Translation

Orygen is a youth mental health organisation based in Melbourne, Australia. Orygen is a unique organisation as it integrates primary through to tertiary (specialist) clinical service delivery, research, and knowledge translation, along with policy and advocacy, service system design and reform. Orygen’s vision is for  all young people to enjoy optimal mental health as they grow into adulthood. A dedicated knowledge translation division focuses on building the capacity of youth mental health systems, services, and workforces to deliver evidence-based prevention and treatment interventions, as an essential strategy to improve the outcomes and experiences of young people in need of mental health support. Several multidisciplinary teams cover a range of knowledge translation functions, including:

- systematic reviewing of peer-reviewed research related to youth mental health for inclusion in Orygen’s online Evidence Finder (Silva et al., 2018);
- design and delivery of resources for the youth mental health workforces;
- design and delivery of training and education through graduate degree programmes at the University of Melbourne, and tailored programmes for youth mental health services, youth services, peer programmes, schools and other organisations that support young people across Australia and internationally;
- consultation and facilitation, including interactive problem-solving, provision of support and quality improvement projects to youth mental health services, as well as direct support and point of care advice to providers (secondary consultation);
- research and evaluation related to workforce development, service improvement and implementation in youth mental health settings.

Each team in the knowledge translation division performs multiple functions, with key differences in funding sources and the services and workforces they support. For instance, providing specialist support to Australian early psychosis services. Across teams, staff roles include clinical educators, implementation facilitators, research and teaching academics, and administrators. Teams collaborate across the division to share knowledge and skills across functions and professional experience (e.g. research, clinical, teaching), and across Orygen with clinical, research, and other areas. This includes close collaboration with communications and lived experience participation specialist areas, which play a key role in supporting knowledge translation activities.

One of the primary foci of Orygen’s knowledge translation division is workforce development, with a dedicated team undertaking activities designed to support the attraction, development and retention of skilled and knowledgeable youth mental health workforces, including clinical and non-clinical professionals. Audiences and participants include a range of professionals whose work involves supporting the mental health and wellbeing of young people, including general practitioners, psychologists, occupational therapists, peer workers, social workers, speech pathologists, dietitians, nurses, psychiatrists, alcohol and another drug workers, teachers, coaches and community volunteers, police members, youth justice officers, legal practitioners, and others. Projects target individuals, teams and whole services including frontline workers, managers and supervisors, administrators and other support staff. While some initiatives aim to support the implementation of evidence-based practices and programmes in specific systems, services or teams, others target the youth mental health workforce more broadly, through self-directed learning by engagement with online resources. All initiatives are designed and delivered with consideration to the unique context of youth mental health.

**Fig. 2:**
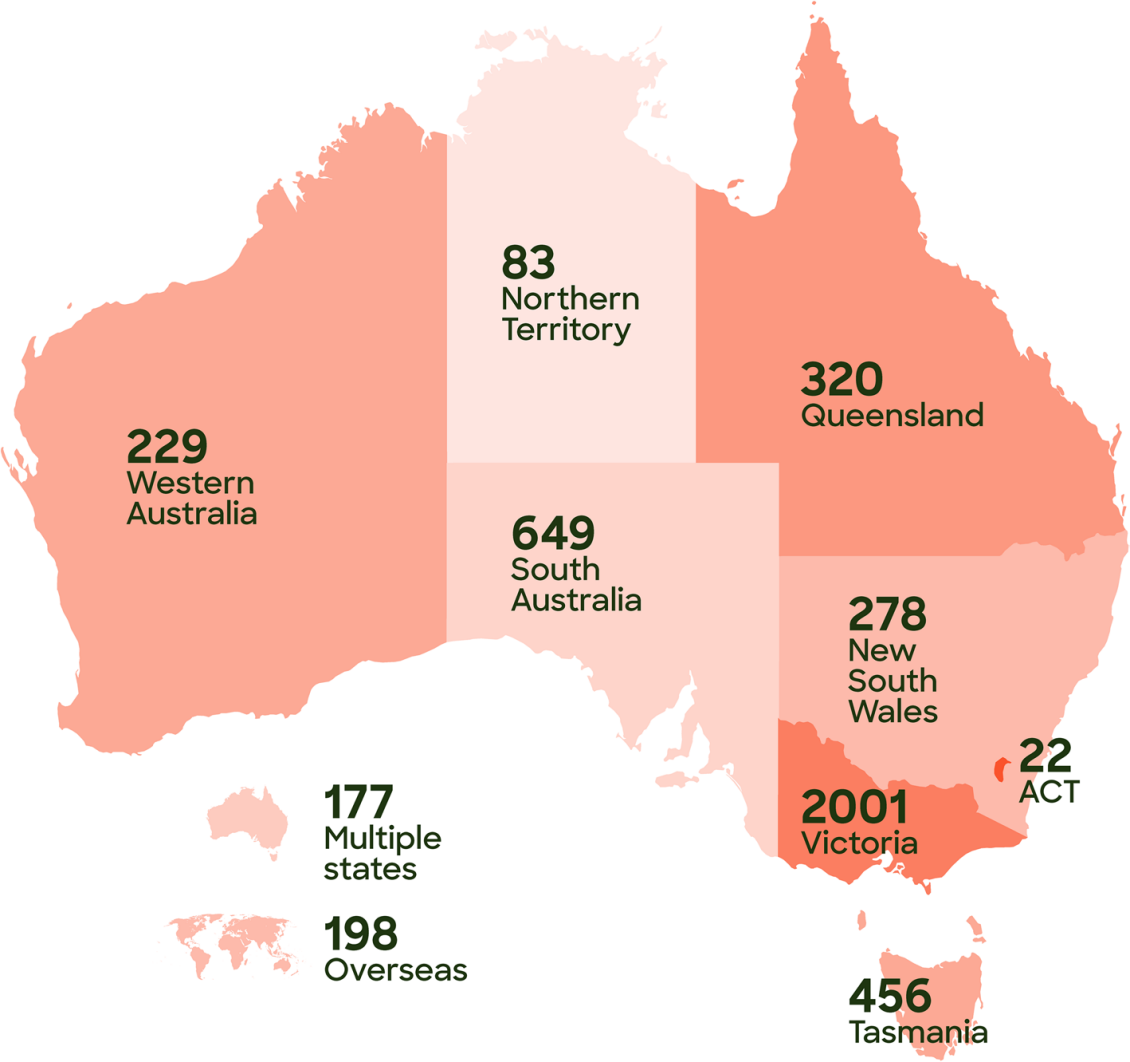
Attendee numbers for face-to-face training delivered by Orygen over 2017–2021

**Fig. 3:**
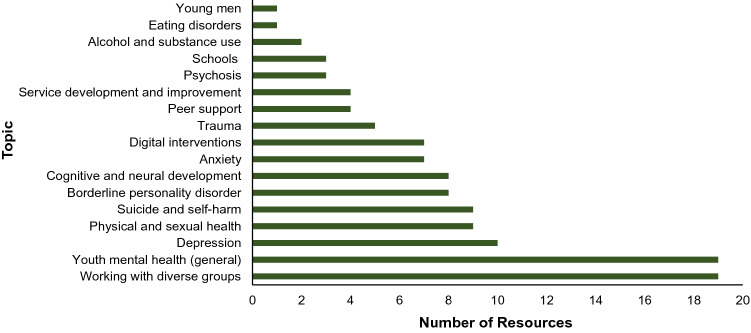
Resources produced by Orygen over 2017–2021

Over the past five years (January 2017–December 2021), the workforce development team at Orygen has delivered professional development programmes to approximately 4400 youth mental health professionals through face-to-face and virtual training, and consultations. Over this period, in-person training was delivered across almost all states and territories of Australia, mostly in Victoria—see Fig. 2. The team also delivered initiatives internationally in Scotland the United States, and Hong Kong. In the same period, the team has produced 119 educational resources, on topics related to youth mental health, including specific psychiatric disorders, such as psychosis, depression, and anxiety, as well as information about digital interventions and supporting mental health online, guidance on service development and delivery, and recommendations on working with diverse groups such as trans and gender diverse young people and refugees and migrants—see Fig. 3. Topics related to general youth mental health include guidance on delivering brief interventions, developing a therapeutic alliance, working with anger, and other skills and knowledge areas related to working with young people experiencing mental ill health. Outputs include written resources like factsheets, clinical practice guides, toolkits, as well as video-based content like webinars, and online training modules that often combine written with game-based tasks and other media like videos to guide the learner through a learning pathway. Over 2017 – 2021 inclusive, Orygen’s workforce development online learning modules were accessed more than 20,000 times, with trauma-informed care, treating complex depression, early intervention for borderline personality disorder, and understanding self-harm among most accessed. Over the period that website data is available, 2018 – 2021 inclusive, online written resources were accessed more than 85,500 times, with guidelines for early psychosis, a brief interventions toolkit and a clinical practice point on trauma-informed care accessed most overall.

## Implementation Science in Workforce Development

Orygen’s knowledge translation division uses a range of frameworks and models from knowledge translation, implementation science and behaviour change theory to inform its programme of work. The Integrated Promoting Action on Research Implementation in Health Services (i-PARIHS) framework provides a guiding framework across activities (Harvey & Kitson, 2016). The i-PARIHS suggests that successful implementation is a function of the relationships between four core elements: (1) the innovation, including knowledge sources, clarity, usability and other characteristics; (2) context, including the local level, inner organisational level and outer health system level; (3) facilitation, including purpose, role and skills and attributes of facilitators; and (4) the recipients, including motivation, goals, time and resources and other factors. Examples of how Orygen’s workforce development team draws on this framework include the collaborative process used to develop educational resources, combining evidence from across academic literature, clinical experiences, and experiences of young people and families. Facilitation forms a major element of the work done by the team, and careful consideration is given to the skills and attributes of team members providing support, with almost all members of the team having clinIcal backgrounds from which they can draw when supporting the implementation of clinical interventions. The team also aims to support positive implementation contexts at the organisational level, through the delivery of resources and consultation on elements such as evaluation and feedback processes, structure and systems, and leadership support. By treating evidence, context and facilitation as equally important in knowledge translation, the i-PARIHS framework supports the workforce development team to understand and leverage a range of factors affecting translation of evidence into practice when designing and delivering workforce development initiatives in complex youth mental health settings.

In addition to drawing on implementation science frameworks, the workforce development team use a range of implementation strategies in its work with services, as defined by the Expert Recommendations for Implementing Change (ERIC) project (Powell et al., 2015). Often this involves applying several strategies, consistent with recommendations from the ERIC project that strategies may be used in isolation or combination. For instance, the team often uses facilitation ‘the process of interactive problem-solving and support that occurs in a context of a recognised need for improvement and a supportive interpersonal relationship’ in combination with other strategies such as conducting educational meetings through the delivery of bespoke training programmes. Flexibly combining implementation strategies and frameworks helps the team to tailor workforce development initiatives depending on a range of considerations, such as amount and quality of available evidence for an innovation, the service setting, audience, or other factors.

## Adapting to COVID-19

The COVID-19 pandemic brought sudden and drastic changes to the mental health system in Australia. Strict public health restrictions (lockdowns) were imposed nationally from March 2020, with the city of Melbourne experiencing significant periods of continuous and cumulative lockdown, including one lasting 112 days reported as among the world’s toughest (Smith, 2020). Young people, families, and clinical and non-clinical professionals were impacted at almost all levels—social, physical, financial, psychological. With little time for adjustment, travel was drastically restricted and face-to-face events and services were limited or no longer available, including the delivery of face-to-face mental health care (Robbins & Midouhas, 2021). Telehealth services, including phone or videoconferencing technology, quickly became the standard tool across mental health settings. The pandemic also generated significant barriers to the delivery of workforce and service development initiatives nationally, with a need to quickly adapt to new models of training and service delivery in the context of increasing pressure on communities’ mental health. For Orygen’s knowledge translation division, this meant a significant and sudden change to the way we worked with youth mental health services and workforces. The following sections discuss key implementation strategies, as defined by the ERIC compilation, that were impacted by pandemic and how the team modified its approach in the face of challenges.

### Conducting Educational Outreach Visits: Pivoting to Virtual Training

**Fig. 4:**
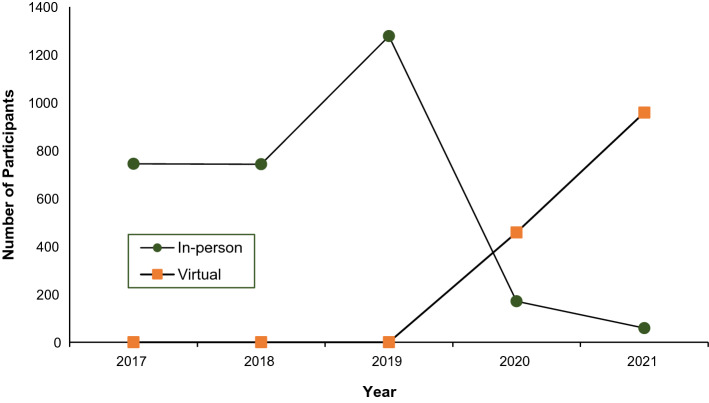
Training delivered by Orygen over 2017–2021

Conducting educational outreach, having ‘a trained person meet with providers in their practice settings to educate providers about the clinical innovation with the intent of changing the provider’s practice’ (Powell et al., 2015) is a key component of the work done by Orygen’s workforce development team and one significantly impacted by the COVID-19 pandemic. Restrictions to in-person events and subsequent banning of travel across Australia led to a rapid and drastic reduction in training delivered in-person in 2020 compared to 2019. In response, the team began delivering virtual workshops via online videoconferencing for the first time. Figure 4 shows the reduction in participants of in-person workshops from 2019 to 2020, along with the emergence of virtual workshops from 2020.

Pivoting to virtual training was a major adjustment for the workforce development team. Notably, virtual workshops were initially more time-intensive to coordinate and prepare, placing limits on how many could be offered at any given time. Online training could also be difficult for services to arrange in terms of technology and space requirements (e.g. access to computers with cameras and microphones for each attendee). Many services who were forced to cancel outreach training chose not to re-schedule to virtual delivery, preferring to wait for face-to-face delivery options. The team noticed that online training could often feel more fatiguing than face-to face training, for both facilitators and participants. Not being able to meet with practitioners in their practice settings also posed a challenge for understanding how practitioners and teams work in context, with clinical educators reflecting that a lot of incidental information is observed and collected from visiting services in-person, including understandings of social and physical environments. The team adjusted to this by collecting information about service environments in other ways, such as in additional preliminary consultation with service leaders, pre-workshop surveys with frontline staff, and spending time during training understanding how teams work together to problem-solve and skill-share.

### Making Training Dynamic

Engaging and dynamic training is a priority for Orygen’s workforce development team, with the pivot from in-person to online delivery posing new challenges for how this was achieved. This strategy aims to ‘vary the information delivery methods to cater to different learning styles and work contexts, and shape the training in the innovation to be interactive’ (Powell et al., 2015). For in-person training, clinical educators design dynamic training through the inclusion of additional written resources, videos, audio files, small group work, larger group discussions, demonstrations, and traditional workshopping techniques using pen and paper. Many of these techniques were maintained in the pivot to virtual training, with some methods adapted to online formats. For instance, using online post-it note and whiteboard tools such as Miro was adopted in some sessions, while the use of live polling tools such as PollEverywhere became standard. This was viewed as a positive of the adjustment to online delivery, with anonymous polling options having benefits for participants sharing opinions on sensitive topics, such as barriers to practice change at service leadership level. Findings new ways to encourage interaction became a focus in training design, with clinical educators reflecting that online learning environments reduced the opportunity for social learning between participants when in large group virtual sessions (Reed et al., 2010). The team adjusted to this by incorporating more small group activities through virtual breakout rooms, where facilitators would virtually move around from group to group, or encourage independent discussions between participants. One aspect of dynamic training missed in virtual environments has been encouraging physical engagement by standing up and moving around the room, which can help to energise training attendees. Facilitators responded to this and the additional fatigue often felt in online training sessions by scheduling more breaks in virtual training than in face-to-face training, and encouraging participants and facilitators to step away from their computers during these times. Implementation science literature on facilitation, including adjusting to online facilitation, helped support the Orygen team in adapting to making virtual training dynamic, providing insights and recommendations from experts (Hartmann et al., 2021).

### Promoting Adaptability

To promote adaptability, the workforce development team ‘identify the ways a clinical innovation can be tailored to meet local needs and clarify which elements of the innovation must be maintained to preserve fidelity’ (Powell et al., 2015). Prior to 2020, the workforce development team would routinely employ this strategy in the design and delivery of workforce development resources and training. Processes for engaging services in workforce development initiatives involve in-depth discussion with service leaders to understand what kind of change a service is aiming to see, what capacity the service has to deliver the change, for instance what type of clinical staff are employed by the service, and what evidence a service has that the change will meet the needs of service users. In Australia, the pandemic prompted a rapid change to delivery of most mental health services from in-person to telehealth care. The workforce development team needed to provide advice on whether clinical innovations could be delivered by telehealth, based on research evidence where available and clinical practice experience with the innovation. To support services, practitioners, and young people adapting to telehealth, the team developed resources about how to effectively deliver and engage with telehealth. Orygen being a research institute means that academic partnerships are a fundamental implementation strategy across workforce development initiatives (Powell et al., 2015). This integration was a key strength during the pandemic, with existing relationships and research staff embedded within the workforce development team enabling rapid literature reviewing for the development of resources, advice provided in consultation, and training content. This helped to support the provision of evidence-based or, where research was emerging, evidence-informed advice about adapting services to telehealth.

## Discussion

The pandemic not only changed how the Orygen workforce development team worked with external services but also it forced us to adapt the way we work as individuals and as a team. Although not formally evaluated, there was a sense of an overall shift in the ‘facilitation intensity’ needed to support staff to implement change (Olmos-Ochoa et al., 2019). Facilitation intensity includes both the activities needed to engage and motivate implementation as well as the psychological impact on the facilitator delivering those activities. There was a perception among the Orygen team that youth mental health service leaders and providers were fatigued by the added demands placed on them by the pandemic, at the individual, service, and system level—and that this impacted on inherent motivation for change and the energy required to engage services in training and development initiatives. For the Orygen team, the impact of lockdowns varied by individual situation, with many team members having to juggle home-schooling and sharing workspaces at home. There was a sense of having to balance a desire for peer support with not wanting to overload colleagues, knowing that others were going through similar challenges. Many reflected on the challenge of reduced organic opportunities for brainstorming and problem-solving with colleagues, and the fatigue associated with virtual meetings. The feedback of remote working increasing number of emails received and this further contributing to stress was common.

While working remotely was seen to create a challenge, the team collectively valued the continued creation of safe spaces to share issues, which moved from in-person team meetings to virtual workshops and phone calls for individual conversations when the team became fatigued by video calls. Team members felt that everyone was willing to work collaboratively to help meet deadlines and complete tasks, with a theme of ‘always having backup’ found in terms of colleagues willing to change their priorities to help others. Regular meetings with team leaders were seen as helpful to keep people on track and adjust workloads and project deadlines as needed. Managers were seen as being responsive to staff needs, placing wellbeing above project outcomes. For instance, delays getting feedback from collaborators on workforce resources placed immense pressure on project deadlines. In many cases, these were adjusted by team leaders, who were acutely aware of the pressure collaborators were under, as many were engaged in frontline mental health care provision or leading changes to service delivery. Empathy and flexibility of managers was viewed as a key factor for helping to support productivity in the long-run. Overall, having a supportive, passionate team, who value collaborative problem-solving, was seen as a major strength.

The pandemic also drew attention to the influence of inner and outer contexts in a new and sudden way, highlighting the importance of organisational factors like access to technology and effective communication, and system factors like policies around access to physical spaces. Using implementation science frameworks helped the Orygen team not only to understand the different levels and factors that can influence implementation and workforce behaviour, but also to share this knowledge with youth mental health practitioners and service leaders to help them understand the pressures they were facing. Perhaps most importantly, implementation science helped the Orygen team build the capacity of youth mental health services to address some of the challenges arising from the pandemic, building awareness of how bridging factors like access to training and facilitation may help to support implementation and behaviour change. The implementation science literature also provided the tools to help services identify other strategies for progressing change, in addition to building knowledge and skills through training and access to educational resources. For instance, providing template implementation action plans to help tailor a generic implementation strategy from the ERIC repository into pragmatic, feasible steps in the local setting (Zbukvic et al., 2022).

Throughout the pandemic, knowledge translation and implementation science frameworks provided structure for the activities and outputs of the workforce development team, they helped give meaning to the challenges experienced in our own work and in the services we support. By prompting the team to consider the influence of multiple factors—including dynamic contexts at the levels of team, organisation, and wider system, service user needs, and capacity of the individuals involved in implementation—implementation science supported the continued delivery of workforce development initiatives in rapidly changing social and physical environments.

## Looking Forward

The pandemic produced uncertainty in multiple areas of our professional and personal lives, over an extended period. In many ways, the Orygen team were accustomed to working with uncertainty, from clinical practice and more broadly from working across emerging fields of youth mental health, knowledge translation and implementation science. For clinical educators on the team, it was easy to see a parallel with clinical work, where coping with uncertainty does not necessarily mean being comfortable, but being able to sit with discomfort and keep in mind shared goals (Connolly, 2022; Marcotte-Beaumier et al., 2020). Using implementation frameworks helped us to maintain shared goals for the team in terms of how we do things (e.g. a goal to build capacity of lived experience advisors that we collaborate with) and the outcomes of our work (e.g. a goal to build the knowledge of youth mental health practitioners attending our training). The culture and relationships within the team were seen as vital for the team’s ability to adapt to the changing pandemic context. Future research on what helped to foster ‘facilitator resilience’ among the workforce development team would be of significant value, given the evidence for the relationship between facilitator resilience and effectiveness (Olmos-Ochoa et al., 2021). Supporting resilience in facilitators may help build and sustain the skilled workforce necessary for continued practice improvement in youth mental health.

One of the most difficult considerations we face as facilitators is knowing when our support will have the most impact for recipients. For the team at Orygen, we have noticed the temptation to work with services that show greater readiness for change, at the service and the team level (Miake-Lye et al., 2020). This becomes a significant factor in our decision to work with a service, given our own capacity limitations as a team, particularly when a service has limited funding for workforce development projects and our contact time is necessarily limited. The pandemic amplified our concerns around this, whereby the tendency to want to work with services who are more progressed in their implementation journey, display stronger culture around innovation and change, or teams who show greater cohesion and capability, may contribute to growing inequity between services where low resources may be contributing to lower readiness. During the pandemic, we heard from services that had lower availability than ever for training and development projects. Healthcare workers experienced immense psychological distress (Smallwood et al., 2021) with child and youth mental health workforces reporting higher workplace stress during the pandemic (McLean & McIntosh, 2021). With youth mental health services expected to experience continued pressure post-pandemic (Chadi et al., 2022), there is a risk that the services who struggle with burnout and recruitment will continue to struggle and in turn have less access to the facilitation that will support their development. Implementation facilitators must be able to adapt their approach to different levels of readiness and implement strategies which build readiness within services. Promising approaches are emerging from the US, such as that described by Watson and colleagues (2022), who used a Readiness Building System for assessing and prioritising readiness, and an Implementation Mapping process to build readiness for the implementation of evidence-based interventions. The Readiness, Resilience and Recovery tool developed out of the same work in response to COVID-19 disruptions, which aims to examine organisational readiness when confronted with a major disruption (Kolodny-Goetz et al., 2021). It is important that such processes can be translated into pragmatic technical guidance which can be used by implementation facilitators more widely.

Perhaps the most significant change to youth mental health care to come out of the pandemic was the rapid shift to digitally delivered services. For the workforce development team, this called new attention to the gap in implementation science theory and practical guidance around ways of working in online environments. Since the pandemic enforced such drastic physical distancing restrictions, consumers are now more likely to have a completely digital interaction with a service—from registering through a web-based form, chatting with an intake worker online, to having telehealth appointments with their psychologist. This holds particular relevance in youth contexts, where the service users are all digital natives, and show interest in digital support for mental health beyond telehealth and the pandemic (Bell et al., 2022). While the pandemic catalysed digital mental health delivery within health systems globally; as a field, digital mental health is nascent in terms of implementation research, which has been described as ‘trial-and-error’ (Graham et al., 2020). Currently, leading implementation science frameworks like the i-PARIHS acknowledge the influence of structures and processes at an organisational level, but do not consider digital realities as environments in which patients access services and interact with clinicians. Nor do they attend to the interrelated social contexts that arise when introducing technology (and often associated data) into work (Sittig & Singh, 2010). Developed to guide the sustained implementation of technology-supported health care interventions, the NASSS (Non-adoption, Abandonment, Scale-up, Spread and Sustain) implementation framework (Greenhalgh et al., 2017) begins to explore these considerations. The seven-domain framework devotes one to technology, addressing not just the technical features of the intervention, but the (i) data or knowledge generated, its trustworthiness and potential role in decision making and patient empowerment; (ii) knowledge and support needed to use the technology; and (iii) issues for sustainability, particularly given the rapid nature of technology turnover and obsolescence. The framework also addresses organisational capacity and readiness for adopting technology-supported change. It falls short, however, of acknowledging that we now live and work in a digital world, which extends through all aspects of inner and outer contexts of implementation, including platforms for service delivery and related policies and procedures to manage interoperability, engagement, safety, confidentiality, and trust in digital systems and spaces.

Based on our experiences, we suggest that the capacity to work with digital tools and within digital environments is now a core element of workforce development across the mental health sector. Technology is increasingly cited as an effective element of the mental health system beyond the pandemic (Bhugra et al., 2017; Pfender, 2020; Torous et al., 2020). This includes youth mental health (Nicholas et al., 2021), where telehealth has been described as ‘an essential new element in the clinicians’ toolkit’ (Hopkins & Pedwell, 2021). Implementation frameworks need to acknowledge the technical skills and experience facilitators require to support workforces and services operating in this emerging aspect of their work. In addition, there are new choices to make as implementation specialists, not just about which implementation and facilitation strategies to use, but which mode of delivery (digital or in-person) will be most effective and most appropriate, with whom, and when. We need implementation research that tests the best way of delivering strategies, not just one strategy against another or against none. Now more than ever, research is needed that will produce practical recommendations on using implementation frameworks in our digital world. This does not mean creating new frameworks but building on those existing, as akin to what has been done with specific health sectors like pharmacy (Moullin et al., 2016). To update and build our toolkits as implementation practitioners, implementation research and practice must respond to our changing context.

## Limitations

This paper reviews the authors' experiences collated from documents and conversations with members of the Orygen workforce development team. As methods did not include formal qualitative or quantitative evaluation, the information presented is descriptive and reflects the individual and shared perspectives of the authors. Future evaluation of specific workforce development initiatives by Orygen may include investigation of experiences and/or impact of virtually delivered implementation strategies during the pandemic.

## Data Availability

Isabel Zbukvic conceived the paper. Material preparation, data collection and analysis were performed by Paula Cruz-Manrique and Isabel Zbukvic with guidance from Caroline Crlenjak. The first draft of the manuscript was written by Isabel Zbukvic and Paula Cruz-Manrique and all authors provided feedback on versions of the manuscript. Jennifer Nicholas and Craig Hamilton provided significant intellectual and scholarly contribution and helped to write a substantially revised version of the manuscript following peer review. All authors read and approved the final manuscript.
